# A comparative analysis of physical activity and cognitive performance in both institutionalized and non-institutionalized older adults, controlling sociodemographic variables

**DOI:** 10.3389/fpsyg.2025.1719468

**Published:** 2025-11-18

**Authors:** Alba Villasán Rueda, Marina Wöbbeking Sánchez, Óscar Fernández-Ballbé, Lizbeth De La Torre, Silvia Wöbbeking Sánchez

**Affiliations:** 1 Facultad de Ciencias de la Salud, UNIE Universidad, Madrid, Spain; 2 Departamento de Personalidad, Evaluación y Tratamiento Psicológico, Facultad de Psicología, Universidad de Salamanca, Salamanca, Spain; 3 Facultad de Ciencias Biomédicas y de la Salud, Universidad Alfonso X (UAX), Madrid, Spain; 4 Facultad de Psicología, CETYS Universidad, Tijuana, Baja California, Mexico; 5 Facultad de Ciencias de la Salud, Universidad Internacional de La Rioja (UNIR), Logroño, Spain

**Keywords:** cognition, institutionalization, physical activity, aging, MoCA

## Abstract

**Introduction:**

This study examined the relationship between physical activity and cognitive performance in older adults living in nursing homes (i.e., those institutionalized for geriatric reasons), taking into account age and educational level as potential confounding factors.

**Methods:**

The sample consisted of 300 participants, 150 of whom were institutionalized in geriatric institutions and 150 of whom lived in community. Cognitive performance was assessed using the Montreal Cognitive Assessment (MoCA), while physical activity levels were measured using the International Physical Activity Questionnaire (IPAQ).

**Results:**

Analysis revealed significant differences in cognitive performance between the two groups, with institutionalized participants achieving lower scores. Furthermore, an interaction effect between institutionalization and physical activity was identified, indicating that exercise is particularly beneficial for the cognitive health of institutionalized individuals.

**Discussion:**

These results imply that physical activity is an especially important protective factor in institutional settings where opportunities for environmental cognitive stimulation may be limited.

## Introduction

Population ageing is one of the most significant demographic phenomena of the 21st century. According to international estimates, the number of people over the age of 60 is expected to double by 2050, presenting enormous health, social and economic challenges (Prince et al., 2015). In this context, maintaining cognitive function in old age has become a priority for scientific research and public policy alike, given that cognitive decline has been associated with reduced quality of life, loss of autonomy, and increased reliance on formal and informal care (Erickson et al., 2022).

The literature has shown that the context of residence is a key factor in cognitive ageing trajectories. Although community living provides more opportunities for social interaction and for environmental stimulation, institutionalization is frequently linked to an elevated risk of cognitive and functional deterioration (Hakimjavadi et al., 2025). Longitudinal and cohort studies have shown that, following admission to long-term care facilities, a significant proportion of older adults experience a rapid deterioration in decision-making ability and autonomy (Anselmo et al., 2025). This phenomenon has been partly explained by the homogenization of routines, the reduction in cognitive stimulus variability, and the limitation of meaningful social interaction.

In light of this, physical activity has been identified as one of the most effective non-pharmacological interventions for mitigating cognitive decline. Various clinical trials and systematic reviews have shown that regular exercise promotes synaptic plasticity and neurogenesis, improves cerebral perfusion and contributes to regulating inflammatory processes related to neurological ageing (Tokgöz and Claassen, 2021; Sampaio et al., 2020). Physical activity has also been consistently shown to be associated with improved performance in episodic memory, executive functions, attention, and processing speed in both healthy adults and those at higher risk of decline (Chen et al., 2025).

In institutional settings in particular, exercise can compensate for a lack of environmental and social stimuli. Rodríguez-Rodríguez et al. (2025). demonstrated that institutionalized older adults who exercise at least three times per week show significantly higher levels of cognitive functioning and experience less deterioration than those who exercise less frequently. This evidence supports the hypothesis that physical activity is a more important protective factor in institutional settings than in the community.

However, despite the growing number of studies on the relationship between exercise and cognition, few studies have analyzed how these variables interact with the residential context or considered how sociodemographic factors, such as age and educational level [linked to the notion of cognitive reserve (Stern, 2012)] influence them. Therefore, it is essential to consider these covariates, as cognitive reserve can modulate the effects of physical activity and institutionalization on cognitive performance.

In the present study, educational level was used as an indicator of cognitive reserve, following Stern’s (2012) model. This approach allowed us to control for individual differences in lifelong cognitive stimulation, which may influence both cognitive performance and the effects of physical activity. Within this framework, the present study aims to provide empirical evidence of the moderating role of physical activity in the relationship between institutionalization and cognition in older adults, while controlling relevant sociodemographic variables. By doing so, the study aims to contribute to closing a gap in current knowledge and generate practical implications to inform the design of intervention programmes that promote cognitive health in institutional settings, where the risk of deterioration is higher and opportunities for stimulation are often more limited.

The originality of this study lies in its main objective: to examine the influence of institutionalization and physical activity on cognitive performance in older adults while controlling for the effects of relevant sociodemographic variables, such as age and educational level.

The specific objectives were to compare the cognitive performance of institutionalized and non-institutionalized older adults while exanimating the effect of physical activity levels on cognition in both groups. Additionally, the study aims to determine whether the relationship between physical activity and cognitive performance differs according to residential context (institutionalization versus non-institutionalization). Finally, the study proposes to evaluate the interaction between institutionalization and physical activity in predicting cognitive performance, while controlling for the participants’ age and educational level.

Based on previous evidence, our hypothesis is that institutionalized older adults will show lower cognitive performance than those living in the community. However, we expect that higher levels of physical activity will mitigate these differences, such that institutionalized individuals with high levels of activity will perform similarly to adults living in the community.

## Method

### Participants

The sample consisted of 300 subjects: 224 women (75%) and 76 men (25%). Of these, 150 lived in institutionalized geriatric centres and 150 lived independently. The participant’s age was between 55 and 99 years old, with an average age of 74.66 years for men and 74.70 years for women, giving a global average age of 74.68 years. Of the institutionalized subjects, 107 were women (71%) and 43 were men (29%), with an average age of 83.17 and an age range of 55–99. The non-institutionalized subjects comprised 117 women (78%) and 33 men (22%), aged between 55 and 84 with an average age of 66.21. There was no significant difference in the proportion of men and women between the institutionalized and community-dwelling groups (*χ*^2^(1) = 1.762, *p* = 0.184). In terms of educational level, 10% of subjects had received no education, placing them close to illiteracy; 50.7% had completed primary education; 20% had completed secondary education, and 19.3% had received university education.

The same inclusion criteria were used for both groups: participants had to be aged 55 or over, show no symptoms of cognitive impairment, and be living in a residential facility or their own home. The absence of cognitive impairment was determined based on participants’ clinical history and reports from healthcare or program staff, rather than MoCA scores, which were collected later as part of the study assessment. Participants with a previous diagnosis of dementia or mild cognitive impairment were excluded.

### Instruments

#### Cognitive variables

Five cognitive indexes were analyzed. The Montreal Cognitive Assessment (Nasreddine et al., 2005) was used to evaluate (1) overall cognitive function. This is a screening test for cognitive impairment which includes tasks of visuospatial and executive functioning, attention, naming, language, abstraction and declarative episodic memory. The Spanish version has adequate internal consistency (*α* = 0.772) and inter-rater reliability (rs = 0.846, *p* < 0.01). The Stroop test (Golden, 2001). was used to assess attentional and inhibitory processes. This test consists of (2) a word reading task, (3) a color naming task and (4) an interference task. The results of the three tasks are transformed into (5) a composite score that offers an interference score after controlling for reading and naming performance. The test exhibits adequate internal consistency for each task [*α* = 0.73–0.82; (Golden, 1975)]. and its content validity has been evaluated in a Spanish sample (Periáñez et al., 2021).

#### Physical activity

The International Physical Activity Questionnaire (IPAQ) (Organización Mundial de la Salud, 2012) was used for this study. This test classifies participants into one of three possible physical activity levels: high, medium or low. It assesses activities related to walking, moderate-intensity exercise and vigorous physical activity in order to do this. The questionnaire has been found to have adequate psychometric properties in the Spanish population (Román Viñas et al., 2013), and is recommended for use in assessing the physical activity levels of older adults (Organización Mundial de la Salud, 2002).

#### Sociodemographic variables

The following sociodemographic variables were collected: gender (female vs. male); age (in years); and educational level (no schooling; primary school; secondary school; or university).

## Procedure

This research was developed in two phases. First, contact was made with various centres to compile the sample, which included older adults living in the community and those residing in institutions. The non-institutionalised group comprised students from the University of Experience at the Pontifical University of Salamanca and the SABIEX program at the Miguel Hernández University in Elche. These university programmes are part of lifelong learning initiatives aimed to benefit older adults. While they differ between universities, they generally do not require specific admission criteria beyond age and primarily aim to provide training opportunities, foster new social networks and encourage intergenerational interaction. It should be noted that they do not seek to obtain an official degree, but rather aim to improve quality of life in old age (Gaede et al., 2020).

The second group, consisting of institutionalised subjects, was recruited from various care homes in the autonomous communities of Castile and León and Valencia (Spain). In order to ensure comparability between groups, efforts were made to ensure that environmental and contextual conditions were as similar as possible. Specifically, all assessments were conducted in quiet, well-lit rooms free from distractions, at similar times, and following the same application protocol and instructions. The tests were administered by previously trained psychologists, who followed standardized procedures to ensure consistency between both environments. Efforts were made to ensure that contextual conditions were similar to those of the first group. The decision to divide the sample between institutionalised and non-institutionalised older adults was made to offer a broader view of the different types of cognitive reserve evaluated, since previous studies had focused only on one of these conditions.

As environmental enrichment is a key factor to the cognitive reserve, the differences between the residential contexts of the two groups provide valuable information for analysis (Gaede et al., 2020; Adam et al., 2013; Chapko et al., 2017). Similarly, the differences in lifestyle between the two groups allow us to anticipate significant variations in physical activity levels, which are a key factor in preserving cognitive reserve (Chen et al., 2025). It is also important to recognize that each environment offers different opportunities for developing life projects and carrying out meaningful activities (Lavrencic et al., 2017; Pettigrew and Soldan, 2019).

In the second phase, each participant was given a series of tests individually, which lasted approximately 75 min. The assessment took place between March and October, with informed consent obtained from all participants in advance. The study strictly adhered to the ethical principles set out in the 2013 revision of the Declaration of Helsinki for this type of research. Note that studies involving human participants were reviewed and approved in accordance with the institutional requirements of the Pontifical University of Salamanca. The Ethics Committee waived the requirement for formal approval because the research included only non-invasive psychological assessments, did not involve potential risks to participants, and was conducted under existing institutional ethical protocols for observational studies. All participants signed a written informed consent form prior to participation, in accordance with the principles of the Declaration of Helsinki (2013 revision). In terms of the inclusion criteria, participants were required to have no cognitive impairment in addition to accepting the established conditions (voluntary participation, legal authorization, and waiver of any financial compensation). Failure to meet any of these criteria resulted in exclusion from the study.

## Data analysis

Descriptive analyses of the main study variables were conducted. Analyses of normality, homoscedasticity and linearity were performed before testing the hypotheses. Normality and homoscedasticity were evaluated for each cognitive variable (being the MoCa, the raw scores obtained from the reading, naming and interference conditions of the Stroop task, and the composite score of the Stroop Task). If these requirements were met, parametric ANCOVAs were performed for each of the cognitive variables; if not, non-parametric ANCOVAs were performed with rank transformation of the dependent variables. This method is based on ranking the dependent variables before computing a classical ANCOVA. Transforming this variable allows to circumvent some potential problems associated with performing a parametric ANCOVA using non-normal raw data, such as loss of statistical power. Moreover, the ranked ANCOVA seems particularly powerful in situations where both homoscedasticity and normality requirements are not met (Leys and Schumann, 2010).

An ANCOVA was performed for each cognitive index, including two fixed factors residence (institutionalized vs. community-dwelling) by physical activity (high, medium and low), controlling for the effects of age and educational level. Participants above or below three standard deviations from the mean in the dependent variable were eliminated. If an interaction effect was obtained in the ANCOVA, *post hoc* tests were performed to determine the differences between institutionalized and community-dwelling individuals at each level of physical activity. Because this approach involves multiple comparisons, there was an increased risk of type I error. To address this, Tukey’s post-hoc corrections were applied. These analyses were conducted using JASP Team (2025) and car (R Core Team, 2021), emmeans (Fox and Weisberg, 2019) and effectsize (Lenth, 2025) packages in R.

## Results

Tables 1, 2 show descriptive statistics for the total sample and for subsamples of individuals living in institutions and in the community.

**Table 1:** Descriptive statistics for qualitative variables.

	Total sample (*n* = 300)		Institutionalized (*n* = 150)		Community dwelling (*n* = 150)	
	*n*	%	*n*	%	*n*	%
Gender						
Women	224	74.7%	107	71.3%	117	78%
Men	76	25.3%	43	28.7%	33	22%
IPAQ						
High	84	28%	26	17.3%	58	38.7%
Medium	118	39.3%	58	38.7%	60	40%
Low	98	32.7%	66	44%	32	21.3%
Educational level						
Non schooled	30	10%	30	20%	0	0%
Primary	152	50.7%	96	64%	56	37.3%
Secondary	60	20%	11	7.3%	49	32.7%
University	58	19.3%	13	8.7%	45	30%

**Table 2:** Descriptive statistics for quantitative variables.

	Total sample (*n* = 300)		Institutionalized (*n* = 150)		Community dwelling (*n* = 150)	
	$\bar{X}$	dt	$\bar{X}$	dt	$\bar{X}$	dt
Age	74.687	11.228	83.167	8.435	66.207	6.088
MoCA	23.127	4.317	20.627	4.388	25.627	2.356
Stroop. Reading	65.373	32.086	40.107	19.742	90.64	19.76
Stroop. Denomination	45.757	22.592	28.71	13.573	62.8	15.972
Stroop. Interference	24.663	12.698	15.433	8.383	33.893	9.042
Stroop. Composite score	−1.632	6.671	−0.939	4.969	−2.325	7.977

Preliminary analyses revealed a significant deviation from normality in the five cognitive indexes. Similarly, Levene’s test revealed a deviation of homoscedasticity in all proposed analyses of variance (MoCA: *F* (5,294) = 14.42, *p* < 0.001; reading: *F* (5,294) = 2.344, *p* = 0.023; color naming: *F* (5,294) = 5.418, *p* < 0.001; interference: *F* (5,294) = 2.576, *p* = 0.027; interference index: *F* (5,294) = 6.763, *p* < 0.001). Consequently, nonparametric ANOVAs were applied using a rank transformation of the dependent variable.

**Figure 1:**
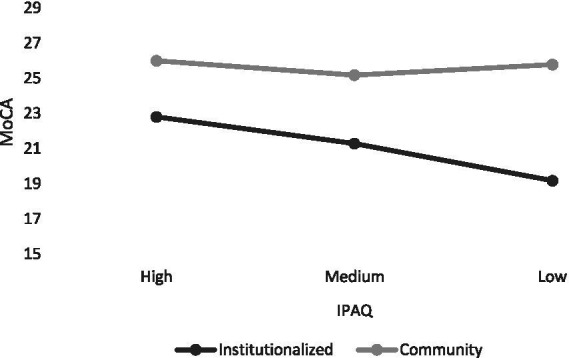
Interaction effect between physical activity and institutionalization on the MoCA.

Since the MoCA did not show any outlier, the ANCOVA was performed using the complete sample (*n* = 300). The analysis revealed significant main effects of age (*F* (1, 292) = 10.1, *p* = 0.001, partial η^2^ = 0.03), educational level (*F* (3, 292) = 17.09, *p* < 0.001, partial η^2^ = 0.15), institutionalization (*F* (1, 292) = 10.54, *p* = 0.006, partial η^2^ = 0.04) and activity level (*F* (2, 292) = 3.46, *p* = 0.032, partial η^2^ = 0.02). Older individuals with lower educational levels and lower physical activity levels, as well as those who were institutionalized, showed significantly lower MoCA scores. The interaction between institutionalization and IPAQ was significant (*F* (2,292) = 5.09, *p* < 0.01, partial η^2^ = 0.03), indicating that the relationship between physical activity and cognition was more pronounced in the institutionalized group (see Figure 1). No significant differences in cognition were observed between the institutionalized group and the community-dwelling group at high levels of physical activity (*F* = (1, 290) = 0.958, *p* = 0.32), but differences were evident at medium (*F* = (1, 290) = 4.105, *p* = 0.043) and low (*F* = (1, 290) = 20.459, *p* < 0.001) levels.

**Figure 2:**
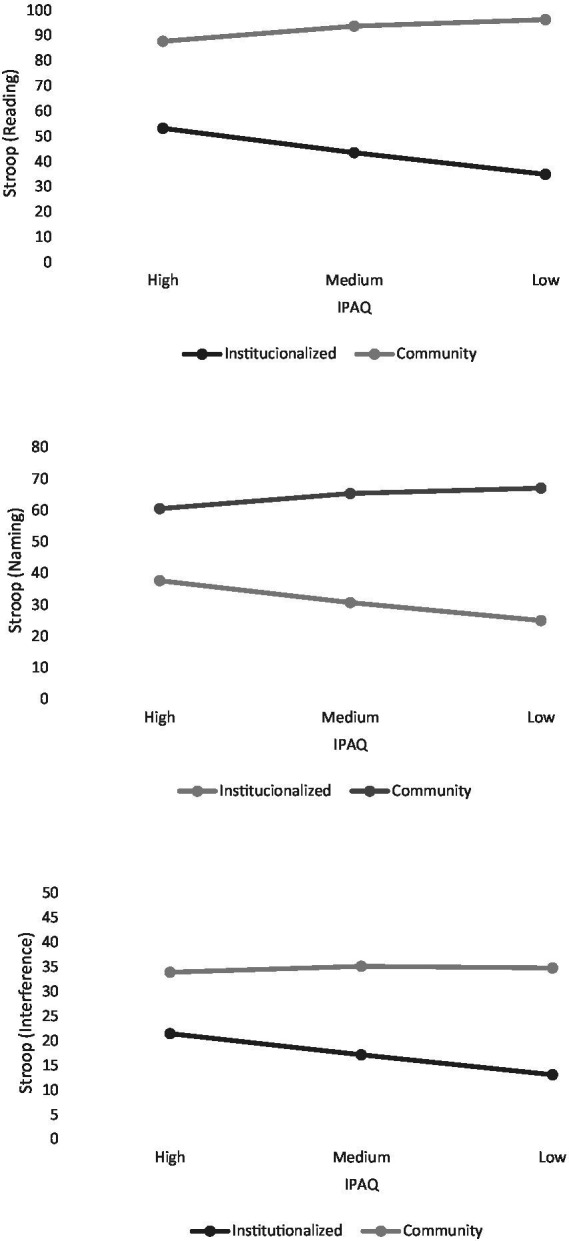
Interaction effect between physical activity and institutionalization on the Stroop test.

ANCOVA analysis of the reading modality of the Stroop test was carried on with the complete sample (n = 300). It revealed that individuals with higher levels of education (*F* (3, 292) = 13.19, *p* < 0.001, partial η^2^ = 0.12) and those living independently (*F* (1, 292) = 123.13, p < 0.001, partial η^2^ = 0.3) achieved significantly higher scores than those institutionalized. However, no main effects were found for age (*F* (1,292) = 2.04, *p* = 0.15, partial η^2^ = 0.007) or level of physical activity. (*F* (2,292) = 1.55, *p* = 0.21, partial η^2^ = 0.001). Analysis of the interaction between the two factors (*F* (2,292) = 9.83, *p* < 0.001, partial η^2^ = 0.06) showed a stronger relationship between task performance and physical activity in institutionalized individuals (see Figure 2). In this case, however, there were significant differences in cognitive performance between institutionalized individuals and those living in the community at high (*F* (1, 290) = 26.074, *p* < 0.001), medium (*F* (1, 290) = 83.028, *p* < 0.001) and low (*F* (1, 290) = 110.532, *p* < 0.001) levels.

In relation to color naming, no significant ouliers were detected (*n* = 300). The ANCOVA showed main effects for educational level (*F* (3, 292) = 14.08, *p* < 0.001, partial η^2^ = 0.013) and institutionalization (*F* (1, 292) = 105.35, *p* < 0.001, partial η^2^ = 0.26), but not for age (*F* (1, 292) = 2.12, *p* = 0.14, partial η^2^ = 0.007) or level of physical activity (*F* (2, 292) = 0.85, *p* = 0.42, partial η^2^ = 0.004). A significant interaction effect was also observed between physical activity level and institutionalization (*F* (1, 292) = 11.5, *p* < 0.001, partial η^2^ = 0.07). Within this interaction (Figure 2), significant differences in cognition were observed between the two groups at high (*F* (1, 290) = 16.729, *p* = 0.001), medium (*F* (1, 290) = 77.883, *p* < 0.001) and low (*F* (1, 290) = 97.781, *p* < 0.001) levels of Physical Activity.

ANCOVA was performed for the Stroop interference modality with a total of 299 participants, as an outlier with a *Z*-score greater than 3 was identified. As in the other modalities, the results indicated that people with higher educational levels (*F* (3, 291) = 14.7, *p* < 0.001, partial η^2^ = 0.13) and those living in a community (*F* (1, 291) = 84.95, p < 0.001, partial η^2^ = 0.23) achieved a significantly higher number of correct answers. A trend for significance was detected for age (*F* (1, 291) = 3.78, *p* = 0.052, partial η^2^ = 0.01). There was no main effect related to physical activity (*F* (2, 292) = 2.27, *p* = 0.109, partial η^2^ = 0.01). Despite the absence of a main effect of physical activity, there was evidence of a significant interaction between physical activity and institutionalization (Figure 2) (*F* (1,291) = 7.36, *p* < 0.001, partial η^2^ = 0.05). *Post hoc* tests revealed significant cognitive differences between institutionalized and community-dwelling individuals at high (*F* (1, 289) = 17.032, *p* < 0.001), medium (*F* (1, 289) = 57.903, p < 0.001) and low (*F* (1, 289) = 77.514, *p* < 0.001) levels of physical activity.

The analysis of the composite interference score from the Stroop test was performed with 297 observations, since three participants were labeled as outliers. The analysis showed only a main effect for institutionalization (*F* (1,289) = 4.69, *p* < 0.03, partial η^2^ = 0.02). There were no main effects for educational level (*F* (3,289) = 0.84, *p* = 0.47, partial η^2^ = 0.008), age (*F* (1,292) = 0.09, *p* = 0.76, partial η^2^ = 0.0003) or level of physical activity (*F* (3,289) = 1.88, *p* = 0.17, partial η^2^ = 0.01). There was also no effect for the interaction between institutionalization and activity level (*F* (2,289) = 1.75, *p* = 0.17, partial η^2^ = 0.01).

In summary, these results show a significant interaction between residential setting and physical activity when predicting cognition in older adults. More specifically, higher levels of physical activity seem especially beneficial in people institutionalized in long-term care facilities. Most of the results were highly significant, suggesting that the findings are robust despite the multiple testing.

## Discussion

The results of this study suggest that institutionalization is associated with poorer cognitive performance in older adults. This finding is consistent with previous research indicating a higher risk of cognitive and functional decline in this type of residential setting (Ben-Shachar et al., 2020; Luppa et al., 2010). However, the present study makes a valuable contribution by demonstrating that physical activity moderates this relationship and acts as a particularly significant protective factor for institutionalized individuals. This finding aligns with the results of Villanueva et al. (2025) research (Sokołowski et al., 2021), which revealed a robust and significant correlation between cognitive decline and physical activity. This suggests that higher levels of physical activity are associated with reduced cognitive decline, indicating that physical activity serves as a protective factor.

This effect can be explained in terms of cognitive reserve (Stern, 2012). Previous studies have investigated changes in cognitive reserve biomarkers according to the type of leisure-time physical activity (aerobic or resistance training), finding significant improvements at 12 weeks compared to baseline measurements. Six-week measurements showed significant decreases in beta-amyloid 1–42 (*p* < 0.01) and homocysteine (*p* < 0.05) (Villanueva et al., 2025).

While older adults living in the community have more opportunities for social interaction and varied cognitive activities, these options are more limited in institutional settings. In this context, physical exercise plays a pivotal role in promoting physiological benefits such as synaptic plasticity, neurogenesis and improved cerebral perfusion (Erickson et al., 2022; Luppa et al., 2010), as well as compensating for the absence of environmental and social stimuli (Kim and Lim, 2022; Rodríguez-Rodríguez et al., 2024).

Similarly, Raffin (Kim and Lim, 2022) has conducted research into the mechanisms through which the benefits of physical interventions occur in the context of cognitive conditions such as Alzheimer’s disease. His findings suggest that physical exercise is widely recognised for its positive impact on brain health, particularly among specific populations such as women with metabolic disorders and Alzheimer’s disease patients with the APOE-ε4 genotype. These populations appear to benefit from physical activity interventions, especially when they are institutionalised in nursing homes.

The interaction patterns observed in the various cognitive tests applied for this study reinforce the hypothesis that physical activity is a more powerful protective factor in contexts of greater vulnerability. The disappearance of differences between groups at high levels of exercise, particularly in the overall MoCA assessment, suggests that regular physical activity may reduce the cognitive gap associated with institutionalization. These results are consistent with recent studies that emphasize the importance of exercise in preventing cognitive decline, regardless of where a person lives (Raffin, 2024; Mao et al., 2023).

This interaction can be better understood through several complementary mechanisms. From the perspective of environmental enrichment, physical activity functions as a form of multisensory stimulation that enhances synaptic plasticity and promotes neurogenesis, compensating for the reduced environmental variability typically found in institutional settings. Moreover, engaging in physical exercise fosters social interaction, shared experiences, and a sense of belonging—factors that are fundamental to maintaining cognitive and emotional well-being in older adults. Motivation also plays a key role: in institutionalized individuals, structured exercise programs can increase self-efficacy, autonomy, and a sense of purpose, buffering the negative impact of routine homogenization and passive lifestyles. Therefore, the interaction between institutionalization and physical activity likely reflects the combined influence of neurobiological, social, and motivational mechanisms that mitigate cognitive decline in contexts of greater vulnerability.

From a practical perspective, the findings of this study highlights the importance of implementing systematic exercise programmes in geriatric institutions. These programmes should not only focus on physical and functional maintenance, but also on strengthening cognitive health. In addition, incorporating multicomponent interventions that combine aerobic, strength and motor coordination exercises could maximize benefits and establish an effective, cost-efficient preventive strategy. This aligns with the findings of Tokgöz and Claassen (2021) who conducted a systematic review concluding that exercise is an effective non-pharmacological treatment for individuals at high risk of Alzheimer’s disease (AD). This treatment can ameliorate AD-related pathological processes, including reducing Aβ load, protecting against hippocampal atrophy, improving cognitive function, stabilizing cholesterol levels, and reducing pro-inflammatory signals. However, exercise appears to delay the onset of AD and improve the quality of life of patients with the condition, regardless of where they live.

Despite the relevance of the findings, this study has several limitations that should be acknowledged. First, its cross-sectional design prevents establishing causal relationships between institutionalization, physical activity, and cognitive performance. Second, other potential confounding factors that could influence both cognitive functioning and physical activity levels—such as comorbidities, nutritional status, depressive symptoms, or medication use—were not controlled for. These factors may interact with the variables studied and partially explain the observed associations. In addition, the assessment of physical activity relied on self-reported measures (IPAQ), which may be subject to recall bias or overestimation. Future studies should employ longitudinal or experimental designs and incorporate objective instruments (e.g., accelerometers), as well as detailed clinical and psychosocial assessments, to better isolate causal pathways and strengthen explanatory models.

From a theoretical point of view, the results support the idea that the relationship between physical activity and cognition is not uniform but rather depends largely on social and environmental context. Therefore, integrative models are needed that simultaneously consider individual (age, educational level, cognitive reserve), behavioral (physical activity, lifestyle habits), and contextual (institutionalization, social support) factors in order to better understand cognitive aging trajectories and design effective interventions.

From a practical perspective, these findings offer valuable guidance for the design of physical activity-based interventions in institutional settings. The results suggest that implementing structured exercise programs can help maintain or even improve cognitive function in institutionalized older adults. Incorporating such programs into the daily routine can increase residents’ autonomy, motivation, and social interaction, mitigating the cognitive decline often associated with institutionalization. In this sense, the study contributes to applied gerontological practice by highlighting the role of regular, multidimensional physical activity as a low-cost, non-pharmacological strategy for promoting cognitive health and overall well-being in long-term care settings.

## Conclusion

This study confirms that institutionalization is a risk factor for cognitive decline in older adults, while physical activity acts as a protective factor, mitigating cognitive differences between institutionalized and community-dwelling individuals. Exercise is especially beneficial for those in institutions, compensating for lower environmental and social stimulation and promoting cognitive performance comparable to independent peers. These findings highlight the importance of implementing structured physical activity programs in geriatric settings that target endurance, strength, and coordination to support cognitive health. Despite the cross-sectional design and limited control of clinical variables, the study provides solid evidence of the interaction between institutionalization and physical activity, suggesting that future longitudinal research with objective exercise measures can strengthen and expand these insights.

## Data Availability

The raw data supporting the conclusions of this article will be made available by the authors, without undue reservation.
